# Account of a Calculus, Taken from the Bladder after Death

**Published:** 1810-09

**Authors:** 


### Account of a Calculus, taken from the Bladder after Death.

[ Laid before the Royal Society of London, by Sir James Earle. ]

THE subject of this Case, Sir Walter Ogilvie, Bart. was an officer in the Army, and at the age of 23, received a blow on his back from the boom of a vessel, which paralyzed the pelvis and lower extremities. During the first two months after the accident, he was obliged to have his water drawn off, and for fourteen months he remained in an horizontal posture; and though he then had recovered the use of the bladder, and of his limbs sufficiently to walk across the room by the help of crutches, and also to ride, when placed on an easy low horse, his health continued many years in a weak and precarious state, while the limbs acquired but little additional strength.

About twenty years after the accident, symptoms were perceived of a stone in the bladder, and it was recommended to him to submit to an operation; but from circumstances it was postponed for eight years, though his health declined, and the irritation and pains in the bladder greatly increased. He now became unable to evacuate his water in an erect position, and the inconvenience increased so much, that at last he could discharge none without standing almost on his head, so as to cause the upper part of the bladder to become lower; and this he was obliged to do frequently, sometimes every ten minutes. At length he came by water to London, and determined to submit to the operation. His sufferings were immense, but the attempt did not succeed: the main body of the calculus was too hard to be broken in pieces, and too large to be brought away, unless by an operation above the os pubis, which was considered as too uncertain and dangerous to hazard even the attempt. In ten days after the operation, he resigned a most singularly miserable existence.

On examination after death, the form of the stone appeared to have been moulded by the bladder; the lower part having been confined by the bony pelvis, took the impression of that cavity, and was smaller than the upper part, which having been unrestricted in its growth, except by the soft parts, was larger, and projected so as to lie on the os pubis. The stone weighed forty-four ounces; the form was eliptical; the periphery on the longer axis was sixteen inches, on the shorter fourteen. The ureters were much much increased in their dimensions and thickness, and were capable of containing a considerable quantity of fluid; they had, in fact, become supplemental bladders, the real bladder being at last nothing more than a painful and difficult conductor of urine, which trickled down in furrows formed by it on the superior surface of the stone. This explained the cause which obliged the patient, when compelled to evacuate urine, to put himself in that posture which made the upper part of the bladder become the lower; by this means a relaxation, or separation, was allowed to take place between the bladder and the stone, so that the ureters had an opportunity of discharging their contents; when the body was erect, their mouths, or valvular openings, must have been closed by the pressure of the abdominal viscera on the bladder, against the stone.

"The disease", says Sir James Earle, "probably originated when the patient was obliged to continue such a length of time on his back, in which position the surface of the water only may be supposed to have been, as it were, decanted, and the bladder seldom, if ever, completely emptied: thus, in a constitution, perhaps naturally inclined to form concretions, the earthy particles subsided, and by attraction soon began to lay the rudiments of a stone, which was not felt above the brim of the pelvis till many years after."

The texture of the stone, upon examination, appeared different from the generality of calculi, to contain more animal matter. Dr. Powell examined its composition by chemical analysis, and found it to consist of the triple phosphate of ammonia and magnesia, with phosphate of lime, mixed with a certain portion of animal matter, which was separated and floated under a membrane-like form, on the solution of the salts in diluted acids.

The calculus agrees with the description given by Fourcroy, and confirms his observations on this species: "Ce sont aussi les concretions urinaires les plus volumineuses de toutes; elles ont depuis le grosseur d'une oeuf jusqu' à une volume qui occupe tout la vessie, en la distendant même considérablement." Hence, it should seem, that similar instances have occurred to this able chemist. "But," says Sir J. Earle, "from my own observation, and from all the information that I have been able to collect, no calculus from the human bladder, of such magnitude, has been hitherto exhibited or described in this country."

